# Synthesis and characterization of bis- and tris-carbonyl Mn(I) and Re(I) PNP pincer complexes

**DOI:** 10.1007/s00706-018-2307-7

**Published:** 2018-10-20

**Authors:** Mathias Glatz, Jan Pecak, Lena Haager, Berthold Stoeger, Karl Kirchner

**Affiliations:** 1 Institute of Applied Synthetic Chemistry, Vienna University of Technology, Getreidemarkt 9/163-AC, 1060 Vienna, Austria; 2 X-ray Center, Vienna University of Technology, Getreidemarkt 9/163-OC, 1060 Vienna, Austria

**Keywords:** Manganese, Rhenium, Pincer complexes, Carbonyl ligands, DFT calculations

## Abstract

**Abstract:**

A series of neutral bis- and cationic tris-carbonyl complexes of the types *cis*-[M(κ^3^*P,N,P*-PNP)(CO)_2_Y] and [M(κ^3^*P,N,P*-PNP)(CO)_3_]^+^ was prepared by reacting [M(CO)_5_Y] (M = Mn, Re; Y = Cl or Br) with PNP pincer ligands derived from the 2,6-diaminopyridine, 2,6-dihydroxypyridine, and 2,6-lutidine scaffolds. With the most bulky ligand PNP^NH^-*t*Bu, the cationic square-pyramidal 16e bis-carbonyl complex [Mn(PNP^NH^-*t*Bu)(CO)_2_]^+^ was obtained. In contrast, in the case of rhenium, the 18e complex [Re(PNP^NH^-*t*Bu)(CO)_3_]^+^ was formed. The dissociation of CO was studied by means of DFT calculation revealing in agreement with experimental findings that CO release from [M(κ^3^*P,N,P*-PNP)(CO)_3_]^+^ is in general endergonic, while for [Mn(κ^3^*P,N,P*-PNP^NH^-*t*Bu)(CO)_3_]^+^, this process is thermodynamically favored. X-ray structures of representative complexes are provided.

**Graphical abstract:**

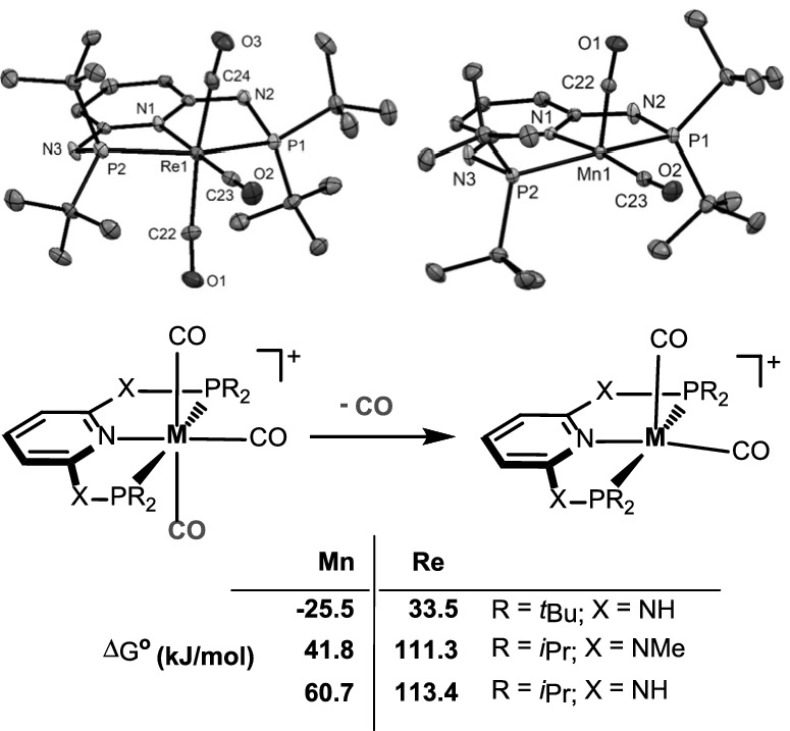

**Electronic supplementary material:**

The online version of this article (10.1007/s00706-018-2307-7) contains supplementary material, which is available to authorized users.

## Introduction

In recent years, manganese pincer complexes, where the metal centers adopt a formal oxidation state of +I, have received considerable importance in the field of homogeneous catalysis [1–6]. In comparison to manganese, rhenium pincer complexes remained comparatively unexplored until very recently but are becoming increasingly important as catalysts for hydrogenation/dehydrogenation reactions [7–10]. The most common ligand architecture is a PNP pincer system featuring an aromatic pyridine backbone with phosphine donors in the two *ortho* positions linked via CH_2_, O, NH, or NMe moieties. In particular, Mn(I) halo and hydride complexes of the type *cis*-[Mn(PNP)(CO)_2_Y] (Y = Cl, Br, H) as shown in Scheme 1 were found to be highly active catalysts in hydrogenation reactions of carbonyl compounds, including CO_2_, as well as nitriles to yield alcohols, formate, and amines, respectively. Moreover, these types of complexes turned out to be also very active catalysts for the opposite process, i.e., dehydrogenation reactions of alcohols to obtain carbonyl compounds. These reactive intermediates are utilized for follow-up reactions such as condensation reactions in the presence of amines to yield functionalized amines, imines, or heterocycles such as pyridines, quinolines, or pyrroles [11, 12].

**Figure:**
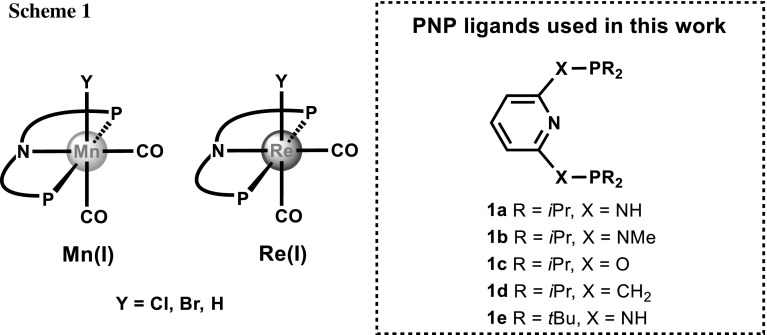

In the present work, we report on the synthesis and reactivity of a series of carbonyl Mn(I) and Re(I) PNP pincer complexes of the types *cis*-[M(κ^3^*P,N,P*-PNP)(CO)_2_Y] (Y = Cl or Br), [M(κ^3^*P,N,P*-PNP)(CO)_3_]^+^, and [M(κ^3^*P,N,P*-PNP)(CO)_2_]^+^ derived from the 2,6-diaminopyridine, 2,6-dihydroxypyridine, and 2,6-lutidine scaffolds.

## Results and discussion

Treatment of the PNP ligands **1a**–**1d** with the carbonyl precursors [Mn(CO)_5_Br] in dioxane afforded complexes of the types *cis*-[Mn(κ^3^*P,N,P*-PNP)(CO)_2_Br] (**2a**–**2d**) and [Mn(κ^3^*P,N,P*-PNP)(CO)_3_]^+^ (**3a**–**3d**) (with Br^−^ as counterion **3aBr**–**3dBr**) in high yields. The outcome of the reaction depends strongly on the reaction temperature (80 °C or 120 °C) and the reaction time (2–18 h) as well as on the nature of the ligand system itself (Scheme 2). At higher temperatures and longer reaction times, the formation of neutral bis-carbonyl complexes is favored, whereas at lower temperatures and shorter reaction times, the formation of cationic tricarbonyl species is preferred. Surprisingly, a tris-carbonyl complex was not obtained with PNP ligand **1c**. It has to be noted that the synthesis of *cis*-[Mn(PNP^NH^-*i*Pr)(CO)_2_Br] (**2a**) [13], *cis*-[Mn(PNP^O^-*i*Pr)(CO)_2_Br] (**2c**) [13], *cis*-[Mn(PNP^CH2^-*i*Pr)(CO)_2_Br] (**2d**) [14], and [Mn(PNP^NH^-*i*Pr)(CO)_3_]^+^ (**3a**) [13, 15] was already described in the literature. These authors, however, reported that with ligand **1a,** they could only obtain an inseparable mixture of **2a** and **3a** [13]. All cationic complexes feature bromide as counterion which can be readily exchanged by other anions, if the bromide complexes are reacted with Ag^+^ salts. This was exemplarily shown for **3a** and **3e**, which upon treatment with AgOTf and AgBF_4_, respectively, yielded complexes **3aOTf** and **3eBF**_**4**_.

**Figure:**
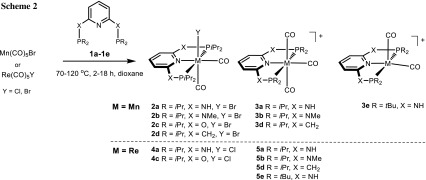

Likewise, ligands **1a**–**1e** reacted with [Re(CO)_5_Y] (Y = Cl or Br) to afford the rhenium(I) complexes *cis*-[Re(κ^3^*P,N,P*-PNP)(CO)_2_Y] (**4a**, **4c**) and [Re(κ^3^*P,N,P*-PNP)(CO)_3_]^+^ (**5a**–**5e**) in high yields (Scheme 2). The synthesis of complex *cis*-[Re(PNP^CH2^-*i*Pr)(CO)_2_Cl] (**4a**) was already described previously [16].

All neutral bis-carbonyl and cationic tricarbonyl complexes, respectively, are orange and off-white air-stable compounds. Selected NMR and IR spectroscopic data are provided in Table 1. In the IR spectrum, complexes **2** and **4** exhibit the two carbonyl stretching frequencies typical for a *cis*-CO arrangement. Complexes **3** and **5** give rise to two or three strong absorption bands typical of a *mer* CO arrangement. In ^13^C{^1^H} NMR, the two or three CO ligands give rise to low field triplets in the range of 238–196 ppm. Due to the quadrupole moment of ^55^Mn (*I* = 5/2), the resonances of the manganese compounds are not always fully resolved giving rise to rather broad signals. Also, in ^31^P{^1^H} NMR spectra, broad singlets are observed.

**Table 1:** Selected ^13^C{^1^H} and ^31^P{^1^H} NMR and IR data of complexes **2**–**6**

Complexes	*δ*_CO_/ppm		*δ*_P_/ppm	ν_CO_/cm^−1^		
[Mn(PNP^NH^-*i*Pr)(CO)_2_Br] (**2a**)			133.6	1925	1819	
[Mn(PNP^NMe^-*i*Pr)(CO)_2_Br] (**2b**)	229.6	222.6	155.6	1929	1853	
[Mn(PNP^O^-*i*Pr)(CO)_2_Br] (**2c**)	228.6	224.3	232.2	1943	1875	
[Mn(PNP^CH2^-*i*Pr)(CO)_2_Br] (**2d**)			85.8	1909	1819	
[Mn(PNP^NH^-*i*Pr)(CO)_3_]^+^ (**3aBr**)	221.0	215.4	133.4	2043	1941	1927
[Mn(PNP^NMe^-*i*Pr)(CO)_3_]^+^ (**3bBr**)	220.3	215.4	156.5	2034	1929	
[Mn(PNP^CH2^-*i*Pr)(CO)_3_]^+^ (**3dBr**)	216.9	207.4	88.3	2028	1937	1916
[Mn(PNP^NH^-*t*Bu)(CO)_2_]^+^ (**3eBr**)	234.9		148.6	1936	1865	
[Mn(PNP^N^-*t*Bu)(CO)_2_] (**6**)	238.2		145.7/142.2	1913	1838	
[Re(PNP^NH^-*i*Pr)(CO)_2_Cl] (**4a**)	208.9	199.2	52.4	1900	1806	
[Re(PNP^O^-*i*Pr)(CO)_2_Br] (**4c**)	197.2	196.6	184.7	1928	1848	
[Re(PNP^NH^-*i*Pr)(CO)_3_]^+^ (**5aBr**)	196.0	191.0	93.8	2045	1926	
[Re(PNP^NMe^-*i*Pr)(CO)_3_]^+^ (**5bBr**)	194.6	190.8	120.9	2045	1945	
[Re(PNP^CH2^-*i*Pr)(CO)_3_]^+^ (**5dCl**)	203.6	193.9	48.6	2041	1936	1916
[Re(PNP^NH^-*t*Bu)(CO)_3_]^+^ (**5eBr**)	233.1	224.0	116.0	2034	1925	1910

In the case of the most bulky PNP ligand **1e**, with [Mn(CO)_5_Br] the cationic 16e bis-carbonyl complex [Mn(PNP^NH^-*t*Bu)(CO)_2_]^+^ (**3e**) was obtained in 95% isolated yield as dark-violet solid (Scheme 2). This is in strong contrast to rhenium, where the 18e complex [Re(PNP^NH^-*t*Bu)(CO)_3_]^+^ (**5e**) was formed. It is interesting to note that also with the analogous 2,6-lutidine-based PNP ligand the cationic 18e complex [Mn(PNP^CH2^-*t*Bu)(CO)_3_]^+^, rather than an unsaturated complex was formed instead [14]. The bromide counterion of **3eBr** could be readily replaced by BF_4_^−^ upon reaction of **3eBr** with AgBF_4_ affording **3eBF**_**4**_. Despite of being coordinatively unsaturated, these complexes are diamagnetic. Coordinatively unsaturated Mn(I) pincer complexes are not uncommon. In fact, several iso-electronic manganese PNP complexes were reported from the groups of Ozereov, Nocera, Boncella, Milstein, Liu, and Jones as shown in Scheme 3 [17–22].

**Figure:**
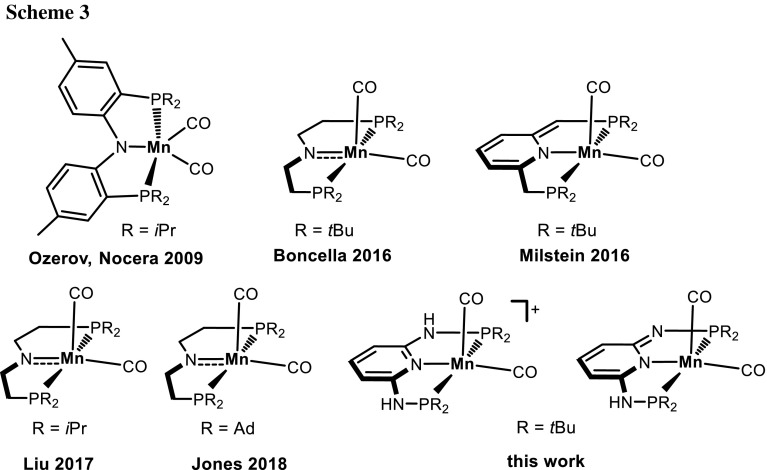

**Fig. 1:**
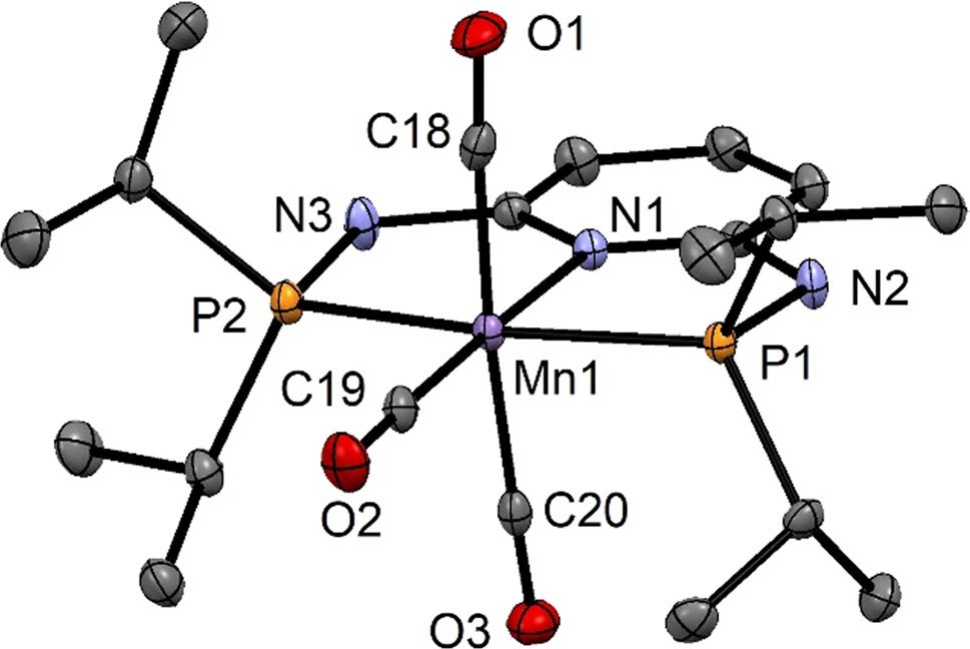
Structural view of [Mn(PNP^NH^-*i*Pr)(CO)_3_]OTf (**3aOTf**) showing 50% thermal ellipsoids (H atoms and triflate counterion omitted for clarity). Selected bond lengths (Å) and bond angles (°): Mn1–C19 1.798(2), Mn1–C20 1.839(2), Mn1–C18 1.857(2), Mn1–N1 2.058(2), Mn1–P1 2.2598(6), Mn1–P2 2.2661(7), C20–Mn1–C18 175.6(1), P1–Mn1–P2 164.84(2)

**Fig. 2:**
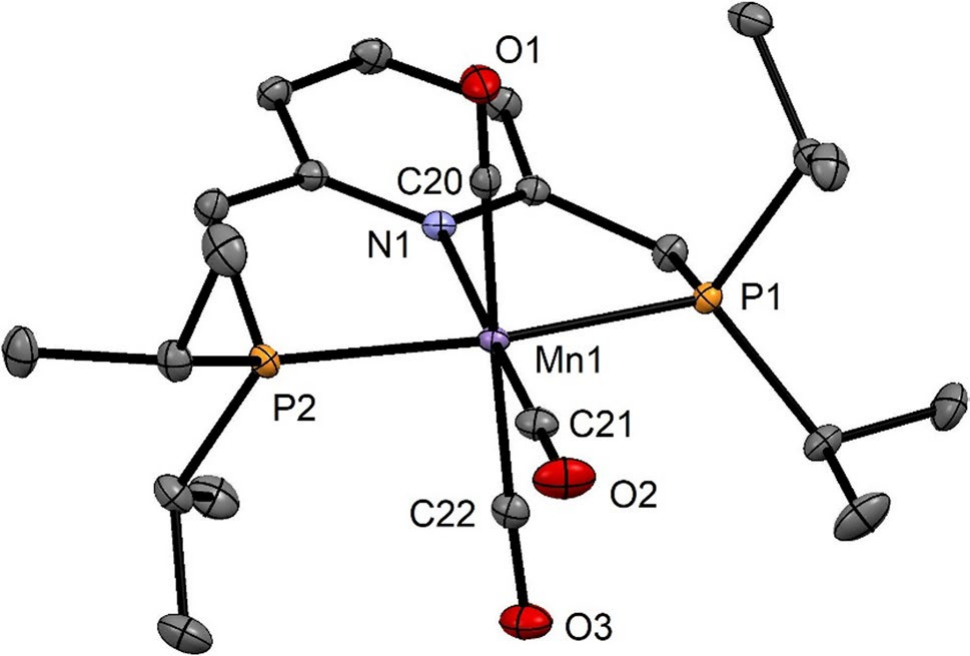
Structural view of [Mn(PNP^CH2^-*i*Pr)(CO)_3_]Br·CH_2_Cl_2_ (**3dBr**·CH_2_Cl_2_) showing 50% thermal ellipsoids (H atoms, solvent, and bromide counterion omitted for clarity). Selected bond lengths (Å) and bond angles (°): Mn1–N1 2.1121(9), Mn1–C20 1.843(1), Mn1–C21 1.792(1), Mn1–C22 1.856(1), Mn1–P2 2.2808(4), Mn1–P1 2.2898(3), C20–Mn1-C22 172.37(5), P1–Mn1–P2 162.60(1)

**Fig. 3:**
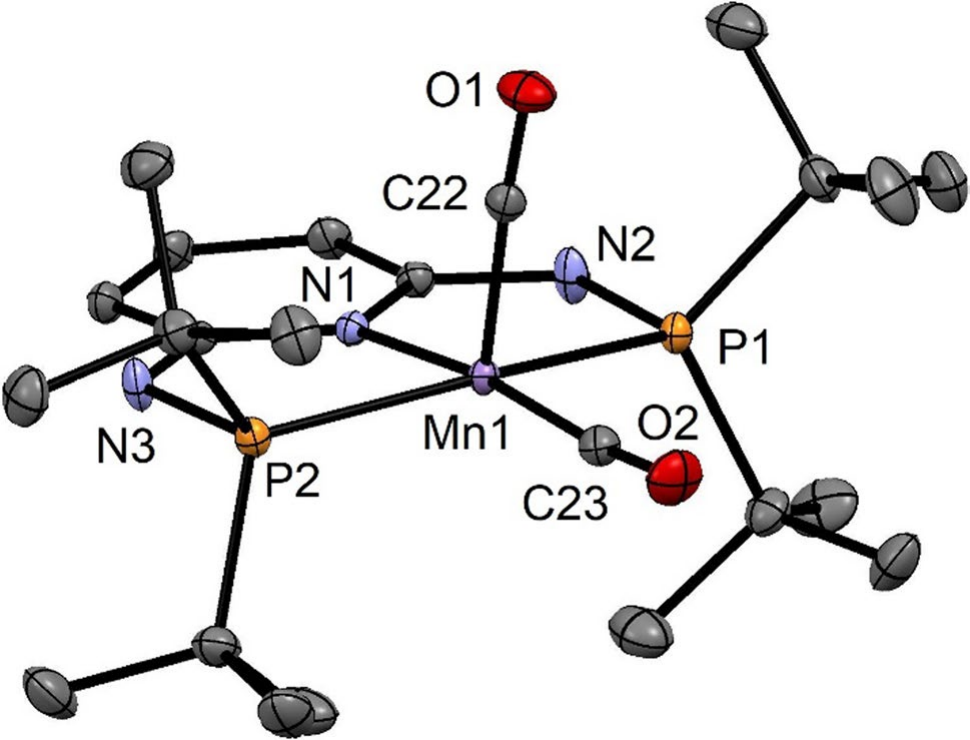
Structural view of [Mn(PNP^NH^-*t*Bu)(CO)_2_]BF_4_ (**3eBF**_**4**_) showing 50% thermal ellipsoids (H atoms and BF_4_^−^ anion omitted for clarity). Selected bond lengths (Å) and bond angles (°): Mn1–C22 1.753(1), Mn1–C23 1.796(1), Mn1–N1 2.033(1), Mn1–P1 2.2929(4), Mn1–P2 2.3047(4), C22–Mn1-C23 83.06(6), C22–Mn1–N1 104.99(6), C23–Mn1–N1 171.95(6), P1–Mn1–P2 162.42(2)

**Fig. 4:**
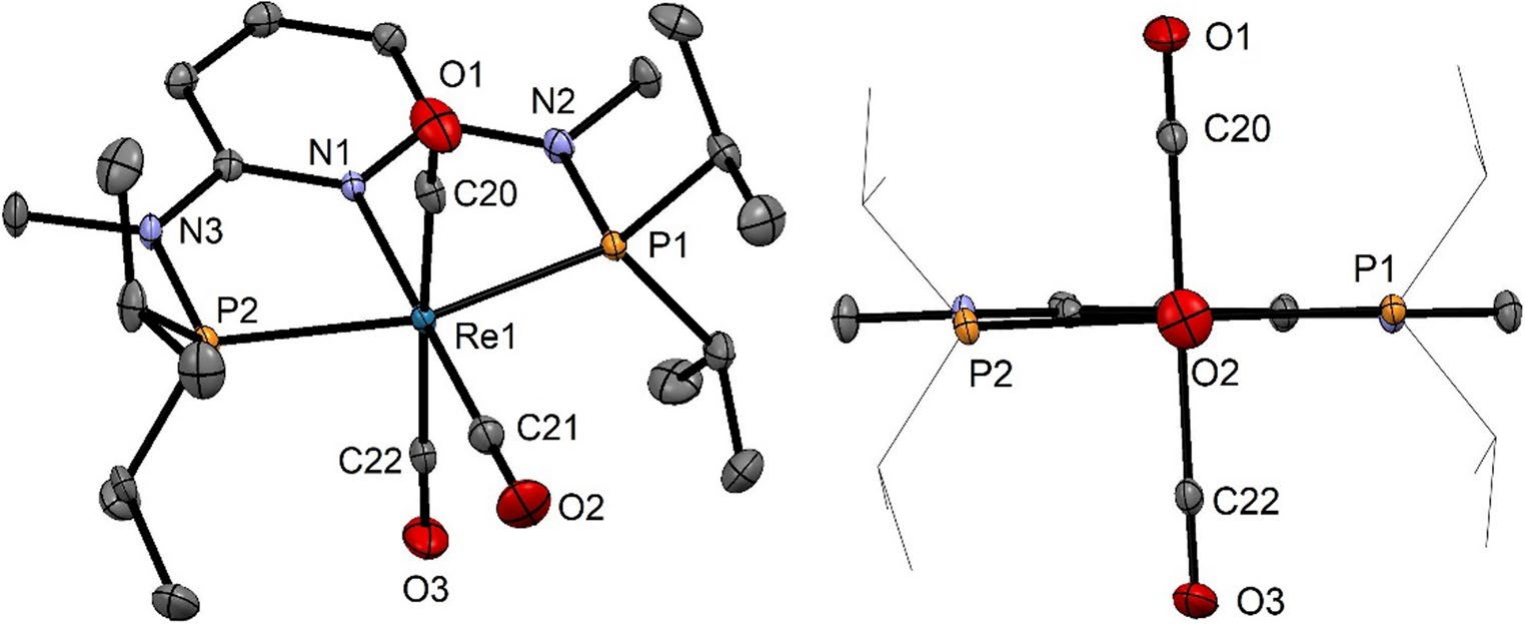
(left) Structural view of [Re(PNP^NMe^-*i*Pr)(CO)_3_]Br·½acetone (**5bBr**·½acetone) showing 50% thermal ellipsoids (H atoms, solvent, and bromide counterion omitted for clarity). (right) Side view across the C–Re–N bond. Selected bond lengths (Å) and bond angles (°): Re1–C20 1.992(3), Re1–C21 1.927(3), Re1–C22 1.970(3), Re1–N1 2.174(2), Re1–P2 2.3822(7), Re1–P1 2.3851(7), C20–Re1–C22 174.7(1), P1–Re1–P2 159.35(2)

**Fig. 5:**
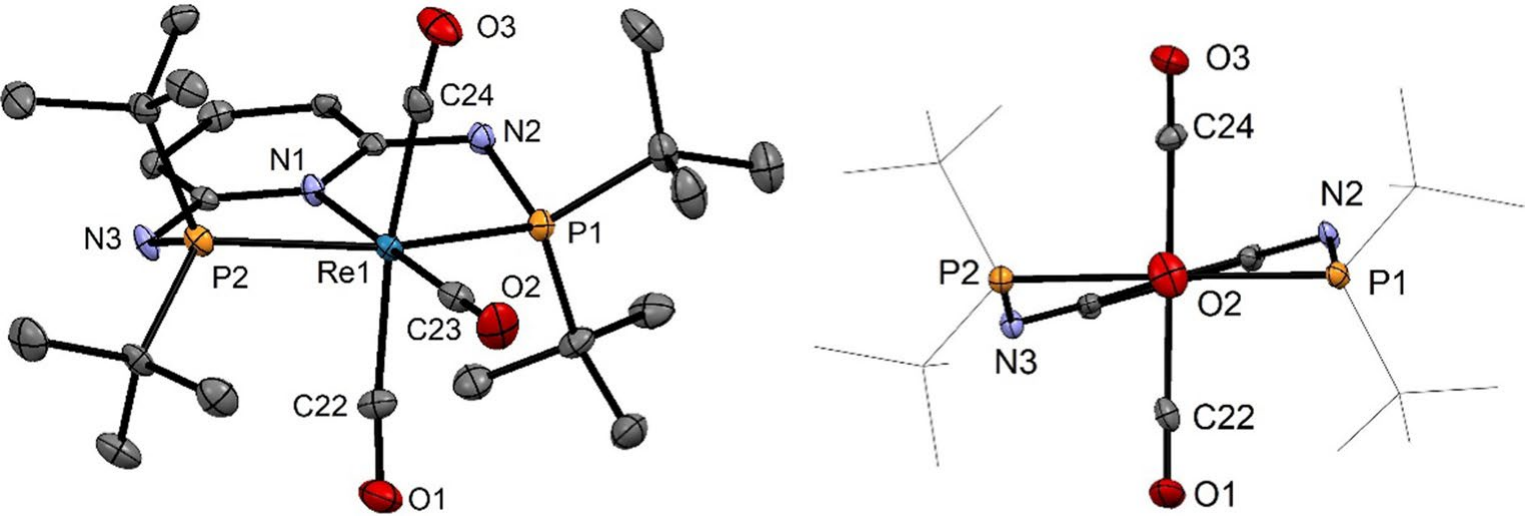
(Left) Structural view of [Re(PNP^NH^-*t*Bu)(CO)_3_]Br (**5eBr**) showing 50% thermal ellipsoids (H atoms and bromide counterion omitted for clarity). (Right) View across the C-Re–N bond. Selected bond lengths (Å) and bond angles (°): Re1–C22 1.980(5), Re1–C23 1.925(5), Re1–C24 1.964(5), Re1–N1 2.186(4), Re1–P1 2.440(1), Re1–P2 2.441(1), C22–Re1–C24 165.1(2), P1–Re1–P2 154.06(4)

In addition to the spectroscopic characterization of all complexes, the molecular structures of complexes [Mn(PNP^NH^-*i*Pr)(CO)_3_]OTf (**3aOTf**), [Mn(PNP^CH2^-*i*Pr)(CO)_3_]Br·CH_2_Cl_2_ (**3dBr**·CH_2_Cl_2_), [Mn(PNP^NH^-*t*Bu)(CO)_2_]BF_4_ (**3eBF**_**4**_), [Re(PNP^NMe^-*i*Pr)(CO)_3_]Br·acetone (**5bBr**·acetone) and [Re(PNP^NH^-*t*Bu)(CO)_3_]Br (**5eBr**) were determined by X-ray crystallography. Structural views are depicted in Figs. 1, 2, 3, 4, 5 with selected bond distances and angles given in the captions. Complexes **3aOTf**, **3dBr**, **5bBr**, and **5eBr** adopt a distorted octahedral geometry around the metal center. The PNP ligand is coordinated to the iron center in a typical tridentate meridional mode, with P-M-P angles between 154.1° and 164.8°. The C_(CO)_–M–C_(CO)_ angles vary between 165.1 and 175.6°. The coordination geometry of Complex **3eBF**_**4**_ is a square pyramid with N(1), P(1), P(2), and C(23) defining the basal plane and C(22) defining the apex.

In the presence of a strong base such as NaH, [Mn(PNP^NH^-*t*Bu)(CO)_2_]^+^ (**3e**) was readily deprotonated to afford the neutral 16e complex [Mn(PNP^N^-*t*Bu)(CO)_2_] (**6**) in 95% isolated yield (Scheme 4). In the ^31^P{^1^H} NMR spectrum, the now inequivalent phosphorous atoms of this complex exhibit a characteristic AB pattern with signals at 145.7 and 142.2 ppm (*J*_*PP*_ = 84.5 Hz). The carbonyl stretches (*ν*_CO_ = 1913, 1838 cm^−1^) are indicative of an increased back-bonding effect relative to the cationic bis-carbonyl complex **3e** (*ν*_CO_ = 1936, 1865 cm^−1^). Recently, Sortais et al. [23] described the deprotonation of complex **2a** to yield [Mn(PNP^N^-*i*Pr)(CO)_3_].

**Figure:**
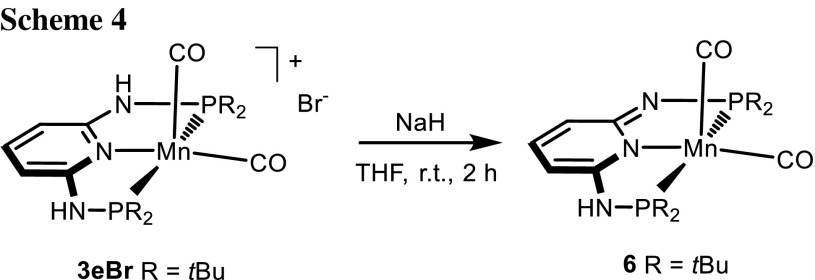

The dissociation of one CO ligand from [M(PNP)(CO)_3_]^+^ (M = Mn, Re) was investigated by means of DFT/B3LYP calculations. The free energies Δ*G*^°^ (in kJ/mol) for the formation of the coordinatively unsaturated complexes [M(PNP)(CO)_2_]^+^ are depicted in Scheme 5. In agreement with experimental findings, the dissociation of CO is in general endergonic ranging from 33.5 to 113.4 kJ/mol. In the case of [Mn(κ^3^*P,N,P*-PNP)(CO)_3_]^+^ (**3e**), this process is thermodynamically favored by − 25.5 kJ/mol. This may be attributed to the bulkiness of the PNP^NH^-*t*Bu ligand, together with the fact that the Mn–C_CO_ bonds are weaker than the Re–C_CO_ bonds [24].

**Figure:**
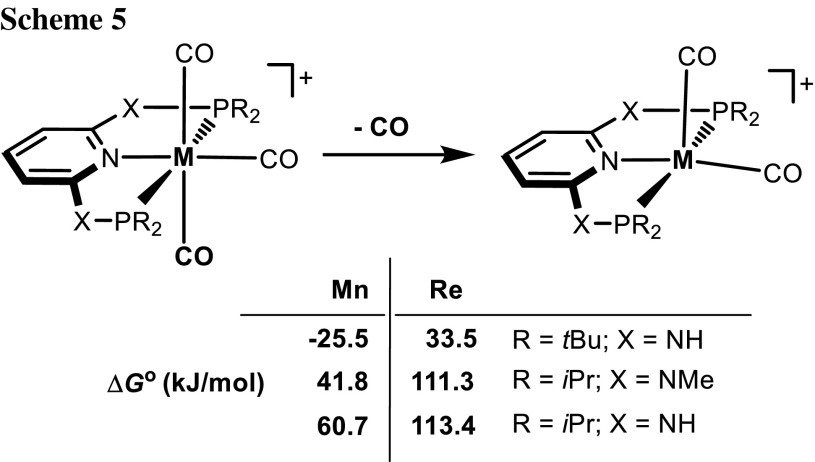

## Conclusion

In sum, we have prepared a series of coordinatively saturated neutral bis- and cationic tris-carbonyl complexes of the types *cis*-[M(κ^3^*P,N,P*-PNP)(CO)_2_Y] and [M(κ^3^*P,N,P*-PNP)(CO)_3_]^+^ by reacting [M(CO)_5_Y] (M = Mn, Re; Y = Cl or Br) with PNP pincer ligands derived from the 2,6-diaminopyridine, 2,6-dihydroxypyridine, and 2,6-lutidine scaffolds. In the case of the most bulky ligand PNP^NH^-*t*Bu, the cationic square-pyramidal 16e bis-carbonyl complex [Mn(PNP^NH^-*t*Bu)(CO)_2_]^+^ was obtained, which in strong contrast to rhenium, where the 18e complex [Re(PNP^NH^-*t*Bu)(CO)_3_]^+^ was formed. The dissociation of CO from [M(κ^3^*P,N,P*-PNP)(CO)_3_]^+^ is typically endergonic ranging from 33.5 to 113.4 kJ/mol. The only exception is [Mn(κ^3^*P,N,P*-PNP^HH^-*t*Bu)(CO)_3_]^+^, where CO dissociation is thermodynamically favorable by − 25.5 kJ/mol as established by DFT/B3LYP calculations. This may be attributed to the bulkiness of the PNP^NH^-*t*Bu ligand, but also due to the fact that Mn–C_CO_ bonds are generally weaker than Re–C_CO_ bonds. Several complexes were also characterized by single crystal X-ray diffraction studies.

## Experimental

All manipulations were performed under an inert atmosphere of argon by Schlenk techniques or in an MBraun inert-gas glovebox. Hydrogen (99.999% purity) was purchased from Messer Austria and used as received. The solvents were purified according to standard procedures [25]. The deuterated solvents were purchased from Aldrich and dried over 4 Å molecular sieves. The pincer ligands PNP^NH^-*i*Pr (**1a**) [26], PNP^NMe^-*i*Pr (**1b**) [27], PNP^O^-*i*Pr (**1c**) [28], PNP^CH2^-*i*Pr (**1d**) [29], and PNP^NH^-*t*Bu (**1e**) [26] were prepared according to the literature. ^1^H, ^13^C{^1^H}, ^19^F{^1^H}, and ^31^P{^1^H} NMR spectra were recorded on Bruker AVANCE-250, 400, and AVANCE-600 spectrometers. ^1^H and ^13^C{^1^H} NMR spectra were referenced internally to residual protio-solvent and solvent resonances, respectively, and are reported relative to tetramethylsilane (*δ* = 0 ppm). ^31^P{^1^H} NMR spectra were referenced externally to H_3_PO_4_ (85%) (*δ* = 0).

### *cis*-[Bromo[*N*^*2*^,*N*^*6*^-bis(diisopropylphosphanyl)pyridine-2,6-diamine](dicarbonyl)manganese(I)], *cis*-[Mn(PNP^NH^-*i*Pr)(CO)_2_Br] (2a, C_19_H_33_BrMnN_3_O_2_P_2_)

Asolution of 341 mg PNP^NH^-*i*Pr (**1a**, 1.2 mmol) and 137 mg [Mn(CO)_5_Br] (1.09 mmol) in 25 cm^3^ dioxane was stirred in a closed vial at 155 °C for 2 h. The solvent was then removed under reduced pressure and 20 cm^3^ of dioxane was added and the mixture was stirred for 2 h. The solvent was then removed under reduced pressure and the solid washed with Et_2_O (3 × 10 cm^3^). The remaining orange solid was dried under vacuum. Yield: 510 mg (91%); ^1^H NMR (250 MHz, CD_2_Cl_2_, 20 °C): *δ* = 7.28 (m, 1H, py^4^), 6.23 (m, 2H, py^3,5^), 5.45 (m, 2H, N*H*), 3.53 (m, 2H, CH), 2.74 (m, 2H, CH), 1.67–1.17 (m, 24H, CH_3_) ppm; ^31^P{^1^H} NMR (101 MHz, CD_2_Cl_2_, 20 °C): *δ* = 135.2 (s, 2P) ppm; IR (ATR): $\bar{\nu }$ = 1925 (*ν*_CO_), 1819 (*ν*_CO_) cm^−1^.

### *cis*-[Bromo[*N*^*2*^,*N*^*6*^-bis(diisopropylphosphanyl)-*N*^*2*^,*N*^*6*^-dimethylpyridine-2,6-diamine](dicarbonyl)manganese(I)], *cis*-[Mn(PNP^NMe^-*i*Pr)(CO)_2_Br] (2b, C_21_H_37_BrMnN_3_O_2_P_2_)

Asolution of 185 mg PNP^NMe^-*i*Pr (**1b**, 0.50 mmol) and 137 mg [Mn(CO)_5_Br] (0.50 mmol) in 15 cm^3^ dioxane was stirred in a closed vessel at 120 °C for 18 h. The solvent was then removed under reduced pressure and the solid washed with 20 cm^3^ *n*-pentane. The yellow powder was dried under vacuum. Yield: 250 mg (89%); ^1^H NMR (600 MHz, acetone-*d*_*6*_, 20 °C): *δ* = 7.57 (t, *J*_*HH*_ = 8.0 Hz, 1H, py^4^), 6.28 (d, *J*_*HH*_ = 8.1 Hz, 2H, py^3,5^), 3.23 (s, 6H, NCH_3_), 3.09 (dt, *J* = 14.0, 7.2 Hz, 2H, CH), 2.98 (dt, *J* = 13.2, 6.8 Hz, 2H, CH), 1.65 (dd, *J* = 14.9, 7.1 Hz, 6H, CH_3_), 1.54 (dd, *J* = 14.7, 7.3 Hz, 6H, CH_3_), 1.49 (dd, *J* = 16.9, 7.1 Hz, 6H, CH_3_), 1.22 (dd, *J* = 13.3, 7.0 Hz, 6H, CH_3_) ppm; ^13^C{^1^H} NMR (151 MHz, acetone-*d*_*6*_, 20 °C): *δ* = 229.6 (CO), 222.9 (CO), 162.7 (vt, *J*_*CP*_ = 10.4 Hz, py^2,6^), 139.3 (s, py^4^), 97.6 (vt, *J*_*CP*_ = 3.1 Hz, py^2,6^), 35.3 (vt, *J*_*CP*_ = 2.6 Hz, NCH_3_), 33.7 (vt, *J*_*CP*_ = 8.9 Hz, CH), 30.2 (vt, *J*_*CP*_ = 11.1 Hz, CH), 21.9 (CH_3_), 19.5 (CH_3_), 17.9 (CH_3_), 17.7 (vt, *J*_*CP*_ = 5.4 Hz, CH_3_) ppm; ^31^P{^1^H} NMR (101 MHz, acetone-*d*_*6*_, 20 °C): *δ* = 155.6 ppm; IR (ATR): $\bar{\nu }$ = 1929 (*ν*_CO_), 1853 (*ν*_CO_) cm^−1^.

### *cis*-[Bromo[2,6-bis[(diisopropylphosphanyl)oxy]pyridine](dicarbonyl)manganese(I)], *cis*-[Mn(PNP^O^-*i*Pr)(CO)_2_Br] (2c, C_19_H_31_BrMnNO_4_P_2_)

Asolution of 137 mg PNP^O^-*i*Pr (**1c**, 0.40 mmol) and 110 mg [Mn(CO)_5_Br] (0.40 mmol) in 10 cm^3^ dioxane was stirred for 2 h at 80 °C. The solvent was removed under reduced pressure and the solid washed with *n*-pentane (3 × 15 cm^3^). The yellow powder was then dried under vacuum. Yield: 197 mg (92%); ^1^H NMR (250 MHz, acetone-*d*_*6*_, 20 °C): *δ* = 7.84 (t, *J*_*HH*_ = 8.1 Hz, 1H, py^4^), 6.86 (d, *J*_*HH*_ = 8.1 Hz, 2H, py^3,5^), 3.61 (m, 2H, CH), 3.03 (m, 4H, CH), 1.56–1.20 (m, 24H, CH_3_) ppm; ^13^C{^1^H} NMR (151 MHz, acetone-*d*_*6*_, 20 °C): *δ* = 228.6 (CO), 224.3 (CO), 163.5 (vt, *J*_*CP*_ = 5.6 Hz, py^2,6^), 142.9 (py^4^), 108.8 (py^3,5^), 27.3 (vt, *J*_*CP*_ = 7.4 Hz, CH), 17.0 (vt, *J*_*CP*_ = 3.6 Hz, CH_3_), 16.9 (vt, *J*_*CP*_ = 4.1 Hz, CH_3_), 16.5 (CH_3_), 15.5 (CH_3_) ppm; ^31^P{^1^H} NMR (101 MHz, acetone-*d*_*6*_, 20 °C): *δ* = 232.2 ppm; IR (ATR): $\bar{\nu }$ = 1943 (*ν*_CO_), 1875 (*ν*_CO_) cm^−1^.

### [[*N*^*2*^,*N*^*6*^-Bis(diisopropylphosphanyl)pyridine-2,6-diamine](tricarbonyl)manganese(I)] trifluoromethanesulfonate, [Mn(PNP^NH^-*i*Pr)(CO)_3_]OTf (3aOTf, C_21_H_33_F_3_MnN_3_O_6_P_2_S)

To a solution of 170 mg PNP^NH^-*i*Pr (**1a**, 0.50 mmol) and 137 mg [Mn(CO)_5_Br] (0.50 mmol) in 15 cm^3^ dioxane 129 mg AgOTf (0.5 mmol) was added and the mixture was stirred at 80 °C for 4 h. Insoluble materials were removed by filtration and the solvent was then removed under reduced pressure. The solid was washed with 15 cm^3^ Et_2_O and 15 cm^3^ *n*-pentane and dried under reduced pressure. Crystals suitable for X-ray diffraction were grown by slow diffusion of *n*-pentane into an acetone solution of **3aOTf**. Yield: 250 mg (89%); ^1^H NMR (400 MHz, acetone-*d*_*6*_, 20 °C): *δ* = 8.28 (m, 2H, N*H*), 7.50 (t, *J*_*HH*_ = 8.0 Hz, 1H, py^4^), 6.55 (d, *J*_*HH*_ = 8.0 Hz, 2H, py^3,5^), 2.93 (m, 4H, CH), 1.53 (dd, *J* = 16.3, 7.0 Hz, 12H, CH_3_), 1.43 (dd, *J* = 17.1, 7.3 Hz, 12H, CH_3_) ppm; ^13^C{^1^H} NMR (151 MHz, acetone-*d*_*6*_, 20 °C): *δ* = 221.0 (CO), 215.4 (CO), 161.0 (vt, *J*_*CP*_ = 7.4 Hz, py^2,6^), 141.0 (py^4^), 99.8 (vt, *J*_*CP*_ = 3.3 Hz, py^2,6^), 30.9 (m, CH), 17.59 (CH_3_), 17.58 (CH_3_) ppm; ^31^P{^1^H} NMR (101 MHz, acetone-*d*_*6*_, 20 °C): *δ* = 133.4 ppm; IR (ATR): $\bar{\nu }$ = 2043 (*ν*_CO_), 1941 (*ν*_CO_), 1927 (*ν*_CO_) cm^−1^.

### [[*N*^*2*^,*N*^*6*^-Bis(diisopropylphosphanyl)-*N*^*2*^,*N*^*6*^-dimethylpyridine-2,6-diamine](tricarbonyl)manganese(I)] bromide, [Mn(PNP^NMe^-*i*Pr)(CO)_3_]Br (3bBr, C_21_H_37_BrMnN_3_O_3_P_2_)

This complex was prepared analogously to **2c** with 185 mg PNP^NMe^-*i*Pr (**1b**, 0.50 mmol) and 137 mg [Mn(CO)_5_Br] (0.50 mmol) as starting materials. Yield: 285 mg (97%); ^1^H NMR (600 MHz, DMSO-*d*_*6*_, 20 °C): *δ* = 7.80 (t, *J*_*HH*_ = 8.0 Hz, 1H, py^4^), 6.47 (d, *J*_*HH*_ = 8.1 Hz, 2H, py^3,5^), 3.36 (m, 4H, CH), 3.17 (s, 6H, NCH_3_), 1.39 (dd, *J* = 17.8, 6.5 Hz, 6H, CH_3_), 1.19 (dd, *J* = 14.3, 6.8 Hz, 6H, CH_3_) ppm; ^13^C{^1^H} NMR (151 MHz, DMSO-*d*_*6*_, 20 °C): *δ* = 220.3 (CO), 215.4 (CO), 162.1 (vt, *J*_*CP*_ = 8.3 Hz, py^2,6^), 142.3 (py^4^), 100.0 (py^2,6^), 35.2 (NCH_3_), 32.5 (vt, *J*_*CP*_ = 12.0 Hz, CH), 18.8 (CH_3_), 18.6 (vt, *J*_*CP*_ = 5.4 Hz, CH_3_) ppm; ^31^P{^1^H} NMR (101 MHz, acetone-*d*_*6*_, 20 °C): *δ* = 156.5 ppm; IR (ATR): $\bar{\nu }$ = 2034 (*ν*_CO_), 1929 (*ν*_CO_) cm^−1^.

### [[2,6-Bis[(diisopropylphosphanyl)methyl]pyridine](tricarbonyl)manganese(I)] bromide, [Mn(PNP^CH2^-*i*Pr)(CO)_3_]Br (3dBr, C_22_H_35_BrMnNO_3_P_2_)

This complex was prepared analogously to **2c** with 172 mg PNP^CH2^-*i*Pr (**1d**, 0.50 mmol) and 137 mg [Mn(CO)_5_Br] (0.50 mmol) as starting materials. Crystals suitable for X-ray diffraction were grown by diffusion of a *n*-pentane into a CH_2_Cl_2_ solution of **3dBr**. Yield: 265 mg (95%); ^1^H NMR of [Mn(PNP^CH2^-*i*Pr)(CO)_3_]OTf (250 MHz, acetone-*d*_*6*_, 20 °C): *δ* = 7.94 (t, *J*_*HH*_ = 7.5 Hz, 1H, py^4^), 7.68 (d, *J*_*HH*_ = 7.4 Hz, 2H, py^3,5^), 4.11 (d, *J*_*HH*_ = 8.5 Hz, 2H, CH_2_), 3.73 (d, *J*_*HH*_ = 8.9 Hz, 2H, CH_2_), 2.82 (dt, *J* = 14.5, 7.3 Hz, 2H, CH), 2.32 (m, 2H, CH), 1.45-1.21 (m, 24H, CH_3_) ppm; ^13^C{^1^H} NMR (151 MHz, DMSO-*d*_*6*_, 20 °C): *δ* = 216.9 (CO), 207.4 (CO), 163.3 (m, py^2,6^), 140.0 (py^4^), 122.6 (py^2,6^), 51.9 (CH_2_), 27.4 (vt, *J*_*CP*_ = 11.4 Hz, CH), 18.7 (d, *J*_*CP*_ = 20.1 Hz, CH), 7.6 (CH_3_) ppm; ^31^P{^1^H} NMR (101 MHz, DMSO-*d*_*6*_, 20 °C): *δ* = 88.3 ppm; IR (ATR): $\bar{\nu }$ = 2028 (*ν*_CO_), 1937 (*ν*_CO_), 1916 (*ν*_CO_) cm^−1^.

### [[*N*^*2*^,*N*^*6*^-Bis(di-*tert*-butylphosphanyl)pyridine-2,6-diamine](dicarbonyl)manganese(I)] bromide, [Mn(PNP^NH^-*t*Bu)(CO)_2_]Br (3eBr, C_23_H_41_BrMnN_3_O_2_P_2_)

This complex was prepared analogously to **2c** with 200 mg PNP-*t*Bu (**1e**, 0.50 mmol) and 137 mg [Mn(CO)_5_Br] (0.50 mmol) as starting materials. The solid was washed twice with 15 cm^3^ THF and 15 cm^3^ *n*-pentane and finally dried under reduced pressure. Yield: 280 mg (95%); ^1^H NMR (250 MHz, DMSO-*d*_*6*_, 20 °C): *δ* = 9.14 (m, 2H, NH), 7.74 (m, 1H, py^4^), 6.63 (d, *J*_*HH*_ = 8.9 Hz, 2H, py^3,5^), 1.36 (m, 36H, CH_3_) ppm; ^13^C{^1^H} NMR (151 MHz, DMSO-*d*_*6*_, 20 °C): *δ* = 235.6 (vt, *J*_*CP*_ = 17.8 Hz, CO), 165.6 (vt, *J*_*CP*_ = 8.6 Hz, py^2,6^), 144.6 (py^4^), 99.6 (m, py^3,5^), 28.4 (CH_3_), 26.7 (C_q_) ppm; ^31^P{^1^H} NMR (101 MHz, DMSO-*d*_*6*_, 20 °C): *δ* = 147.6 ppm; IR (ATR): $\bar{\nu }$ = 1936 (*ν*_CO_), 1865 (*ν*_CO_) cm^−1^.

### [[*N*^*2*^,*N*^*6*^-Bis(di-*tert*-butylphosphanyl)pyridine-2,6-diamine](dicarbonyl)manganese(I)] tetrafluoroborate, [Mn(PNP^NH^-*t*Bu)(CO)_2_]BF_4_ (3eBF_4_, C_23_H_41_BF_4_MnN_3_O_2_P_2_)

Asolution of 200 mg PNP-*t*Bu (**1e**, 0.50 mmol) and 137 mg [Mn(CO)_5_Br] (0.50 mmol) in 15 cm^3^ dioxane was stirred at 80 °C for 4 h. A dark-violet solid was formed which was filtered on a glass frit, washed with 15 cm^3^ Et_2_O and dried under vacuum. To a solution of this violet powder in 10 cm^3^ acetone, 98 mg AgBF_4_ (0.5 mmol) was added and the mixture was stirred for 1 h. The precipitate was removed by filtration over Celite and the solvent was then removed under reduced pressure. The solid was washed with 15 cm^3^ Et_2_O and 15 cm^3^ *n*-pentane and finally dried under vacuum. Crystals suitable for X-ray diffraction were grown by slow diffusion of *n*-pentane into an acetone/EtOH (1:1) solution of **3eBF**_**4**_. Yield: 245 mg (82%); ^1^H NMR (250 MHz, acetone-*d*_*6*_, 20 °C): *δ* = 8.49 (m, 2H, N*H*), 7.76 (t, 1H, *J*_HH_ = 8.0 Hz, py^4^), 6.80 (d, *J*_*HH*_ = 8.0 Hz, 2H, py^3,5^), 1.36 (m, 36H, CH_3_) ppm; ^13^C{^1^H} NMR (151 MHz, acetone-*d*_*6*_, 20 °C): *δ* = 234.9 (vt, *J*_*CP*_ = 17.2 Hz, CO), 165.2 (vt, *J*_*CP*_ = 8.3 Hz, py^2,6^), 144.4 (py^4^), 99.8 (vt, *J*_*CP*_ = 3.2 Hz, py^3,5^), 39.7 (vt, *J*_*CP*_ = 8.6 Hz, C_q_), 27.7 (vt, *J*_*CP*_ = 2.0 Hz, CH_3_) ppm; ^31^P{^1^H} NMR (101 MHz, acetone-*d*_*6*_, 20 °C): *δ* = 148.6 (s, 2P) ppm; IR (ATR): $\bar{\nu }$ = 1936 (*ν*_CO_), 1865 (*ν*_CO_) cm^−1^.

### *cis*-[Chloro[2,6-bis[(diisopropylphosphanyl)oxy]pyridine](dicarbonyl)rhenium(I)], *cis*-[Re(PNP^O^-*i*Pr)(CO)_2_Cl] (4c, C_19_H_31_ClNO_4_P_2_Re)

A solution of 136 mg PNP^O^-*i*Pr (**1c**, 0.4 mmol) and 144 mg [Re(CO)_5_Cl] (0.4 mmol) in 15 cm^3^ dioxane was stirred in a closed vessel at 120 °C for 18 h. The solvent was removed under reduced pressure. The obtained solid was washed with 10 cm^3^ Et_2_O and 20 cm^3^ *n*-pentane and dried under vacuum. Yield: 228 mg (92%); ^1^H NMR (600 MHz, acetone-*d*_*6*_, 20 °C): *δ* = 7.76 (t, *J*_*HH*_ = 8.1 Hz, 1H, py^4^), 6.80 (d, *J*_*HH*_ = 8.1 Hz, 2H, py^3,5^), 3.59 (m, 2H, CH), 2.89 (m, 2H, CH), 1.29 (dd, *J* = 12.9, 7.0 Hz, 6H, CH_3_), 1.23 (m, 12H, CH_3_), 1.09 (dd, *J* = 15.1, 7.2 Hz, 6H, CH_3_) ppm; ^13^C{^1^H} NMR (151 MHz, acetone-*d*_*6*_, 20 °C): *δ* = 203.6 (CO), 193.9 (CO), 163.2 (vt, *J*_*CP*_ = 3.7 Hz, py^2,6^), 143.4 (py^4^), 102.8 (vt, *J*_*CP*_ = 1.9 Hz, py^3,5^), 27.9 (vt, *J*_*CP*_ = 12.0 Hz, CH), 17.6 (vt, *J*_*CP*_ = 5.3 Hz, CH), 17.1 (vt, *J*_*CP*_ = 4.6 Hz, CH_3_), 16.7 (CH_3_), 15.0 (CH_3_) ppm; ^31^P{^1^H} NMR (101 MHz, acetone-*d*_*6*_, 20 °C): *δ* = 184.7 ppm; IR (ATR): $\bar{\nu }$ = 1928 (*ν*_CO_), 1848 (*ν*_CO_) cm^−1^.

### [[*N*^*2*^,*N*^*6*^-Bis(diisopropylphosphanyl)pyridine-2,6-diamine](tricarbonyl)rhenium(I)] bromide, [Re(PNP^NH^-*i*Pr)(CO)_3_]Br (5aBr, C_20_H_33_BrN_3_O_3_P_2_Re)

Asolution of 206 mg PNP-*i*Pr (**1a**, 0.6 mmol) and 244 mg [Re(CO)_5_Br] (0.6 mmol) in 10 cm^3^ dioxane was stirred for 2 h at 80 °C. The solvent was then removed under reduced pressure and the solid was washed with *n*-pentane (3 × 15 cm^3^). The colorless powder was finally dried under vacuum. Yield: 394 mg (95%); ^1^H NMR (250 MHz, DMSO-*d*_*6*_, 20 °C): *δ* = 9.21 (m, 2H, NH), 7.54 (t, *J*_*HH*_ = 8.0 Hz, 1H, py^4^), 6.46 (d, *J*_*HH*_ = 8.1 Hz, 2H, py^3,5^), 2.68 (m, 4H, CH), 1.35 (dd, *J* = 17.2, 6.9 Hz, 12H, CH_3_), 1.21 (dd, *J* = 17.6 Hz, 7.3 Hz, 12H, CH_3_) ppm; ^13^C{^1^H} NMR (63 MHz, DMSO-*d*_*6*_, 20 °C): *δ* = 196.0 (CO), 191.0 (vt, *J*_*CP*_ = 9.2 Hz, CO), 162.3 (vt, *J*_*CP*_ = 6.0 Hz, py^2,6^), 141.8 (py^4^), 99.4 (py^3,5^), 31.4 (vt, *J*_*CP*_ = 15.9 Hz, CH), 18.9 (CH_3_) ppm; ^31^P{^1^H} NMR (101 MHz, DMSO-*d*_*6*_, 20 °C): *δ* = 93.8 ppm; IR (ATR): $\bar{\nu }$ = 2045 (*ν*_CO_), 1926 (*ν*_CO_) cm^−1^.

### [[*N*^*2*^,*N*^*6*^-Bis(diisopropylphosphanyl)-*N*^*2*^,*N*^*6*^-dimethylpyridine-2,6-diamine](tricarbonyl)rhenium(I)] bromide, [Re(PNP^NMe^-*i*Pr)(CO)_3_]Br (5bBr, C_22_H_37_BrN_3_O_3_P_2_Re)

This complex was prepared analogously to **5aBr** with 222 mg PNP^NMe^-*i*Pr (**1b**, 0.60 mmol) and 244 mg [Re(CO)_5_Br] (0.60 mmol) as starting materials. Crystals suitable for X-ray diffraction were grown by slow diffusion of *n*-pentane in an acetone solution of **5bBr**. Yield: 405 mg (94%); ^1^H NMR (600 MHz, acetone-*d*_*6*_, 20 °C): *δ* = 7.90 (t, *J*_*HH*_ = 8.3 Hz, 1H, py^4^), 6.65 (d, *J*_*HH*_ = 8.3 Hz, 2H, py^3,5^), 3.39 (m, 6H, NCH_3_), 2.94 (m, 4H, CH), 1.46 (dd, *J* = 19.8 Hz, 6.9 Hz, 12H, CH_3_), 1.22 (dd, *J* = 19.8 Hz, 6.9 Hz, 12H, CH_3_) ppm; ^13^C{^1^H} NMR (151 MHz, acetone-*d*_*6*_, 20 °C): *δ* = 194.6 (CO), 190.8 (t, *J*_*CP*_ = 9.3 Hz, CO), 163.1 (vt, *J*_*CP*_ = 7.2 Hz, py^2,6^), 142.0 (s, py^4^), 100.2 (vt, *J*_*CP*_ = 2.6 Hz, py^3,5^), 35.4 (NCH_3_), 32.25 (vt, *J*_*CP*_ = 15.2 Hz, CH), 19.2 (vt, *J*_*CP*_ = 4.6 Hz, CH_3_), 17.9 (CH_3_) ppm; ^31^P{^1^H} NMR (101 MHz, acetone-*d*_*6*_, 20 °C): *δ* = 120.9 ppm; IR (ATR): $\bar{\nu }$ = 2045 (*ν*_CO_), 1925 (*ν*_CO_) cm^−1^.

### [[2,6-Bis[(diisopropylphosphanyl)methyl]pyridine](tricarbonyl)rhenium(I)] chloride, [Re(PNP^CH2^-*i*Pr)(CO)_3_]Cl (5dCl, C_22_H_35_ClNO_3_P_2_Re)

This complex was prepared analogously to **5aBr** with 136 mg PNP^CH2^-*i*Pr (**1d**, 0.4 mmol) and 144 mg [Re(CO)_5_Cl] (0.4 mmol) as starting materials. Yield: 251 mg (97%); ^1^H NMR (600 MHz, DMSO-*d*_*6*_, 20 °C): *δ* = 8.02 (t, *J*_*HH*_ = 7.7 Hz, 1H, py^4^), 7.66 (d, *J*_*HH*_ = 7.8 Hz, 2H, py^3,5^), 4.26 (m, 4H, CH_2_), 2.60 (m, 4H, CH), 1.24 (dd, *J* = 16.2, 7.0 Hz, 12H, CH_3_), 1.12 (dd, *J* = 16.4 Hz, 7.2 Hz, 12H, CH_3_) ppm; ^13^C{^1^H} NMR (151 MHz, DMSO-*d*_*6*_, 20 °C): *δ* = 198.2 (CO), 193.8 (vt, *J*_*CP*_ = 8.3 Hz, CO), 165.0 (vt, *J*_*CP*_ = 3.0 Hz, py^2,6^), 140.6 (py^4^), 122.3 (vt, *J*_*CP*_ = 4.6 Hz, py^3,5^), 42.1 (vt, *J*_*CP*_ = 13.9 Hz, CH_2_), 27.7 (vt, *J*_*CP*_ = 14.3 Hz, CH_3_), 18.9 (vt, *J*_*CP*_ = 13.3 Hz, CH_3_) ppm; ^31^P{^1^H} NMR (101 MHz, DMSO-*d*_*6*_, 20 °C): *δ* = 48.6 ppm; IR (ATR): $\bar{\nu }$ = 2041 (*ν*_CO_), 1936 (*ν*_CO_), 1916 (*ν*_CO_) cm^−1^.

### [[*N*^*2*^,*N*^*6*^-Bis(di-*tert*-butylphosphanyl)pyridine-2,6-diamine](dicarbonyl)rhenium(I)] bromide, [Re(PNP^NH^-*t*Bu)(CO)_3_]Br (5eBr, C_24_H_41_BrN_3_O_3_P_2_Re)

This complex was prepared analogously to **5aBr** with 199 mg PNP^NH^-*t*Bu (**1e**, 0.50 mmol) and 203 mg [Re(CO)_5_Br] (0.50 mmol) as starting materials. Crystals suitable for X-ray diffraction were grown by slow diffusion of *n*-pentane into an acetone solution of **5eBr**. Yield: 360 mg (96%); ^1^H NMR (250 MHz, DMSO-*d*_*6*_, 20 °C): *δ* = 9.04 (m, 2H, NH), 7.58 (t, *J*_*HH*_ = 7.9 Hz, 1H, py^4^), 6.63 (d, *J*_*HH*_ = 7.9 Hz, 2H, py^3,5^), 1.42 (m, 36H, CH_3_) ppm; ^13^C{^1^H} NMR (151 MHz, DMSO-*d*_*6*_, 20 °C): *δ* = 197.2 (CO), 196.6 (t, *J*_*CP*_ = 8.5 Hz, CO), 162.8 (py^2,6^), 142.4 (py^4^), 100.1 (py^3,5^), 42.0 (vt, *J*_*CP*_ = 10.9 Hz, C_q_), 29.6 (vt, *J*_*CP*_ = 2.4 Hz, CH_3_) ppm; ^31^P{^1^H} NMR (101 MHz, DMSO-*d*_*6*_, 20 °C): *δ* = 116.0 (s, 2P) ppm; IR (ATR): $\bar{\nu }$ = 2034 (*ν*_CO_), 1925 (*ν*_CO_), 1910 (ν_CO_) cm^−1^.

### [[*N*-(di-*tert*-Butylphosphanyl)-6-[(di-*tert*-butylphosphanyl)-*λ*^*2*^-azanyl]pyridine-2-amine](dicarbonyl)manganese(I)], [Mn(PNP^HN^-*t*Bu)(CO)_2_] (6, C_23_H_40_MnN_3_O_2_P_2_)

To a suspension of 118 mg [Mn(PNP^NH^-*t*Bu)(CO)_2_]Br (**5eBr**, 0.20 mmol) in 15 cm^3^ THF, 11 mg NaH (0.46 mmol) was added. The suspension turned deep blue after 10 min and was stirred for an additional 2 h. Insoluble materials were removed by filtration over Celite. The solvent was then removed under reduced pressure. The crude product was redissolved in 20 cm^3^ *n*-pentane, filtered over Celite, and the solvent was removed under vacuum to afford **5eBr** as blue solid. Yield: 96 mg (95%); ^1^H NMR (250 MHz, C_6_D_6_, 20 °C): *δ* = 6.91 (t, *J*_*HH*_ = 7.4 Hz, 1H, py^4^), 6.79 (d, *J*_*HH*_ = 8.4 Hz, 1H, py^3^), 5.13 (d, *J*_*HH*_ = 6.9 Hz, 1H, py^5^), 4.27 (d, *J*_*HH*_ = 6.7 Hz, 1H, NH), 1.36 (d, *J*_HP_ = 13.0 Hz, 18H, CH_3_), 0.94 (d, *J*_HP_ = 13.7 Hz, 18H, CH_3_) ppm; ^13^C{^1^H} NMR (151 MHz, C_6_D_6_, 20 °C): *δ* = 238.2 (vt, *J*_*CP*_ = 16.2 Hz, CO), 174.6 (dd, *J*_*CP*_ = 8.4, 2.8 Hz, py^2^), 162.0 (dd, *J*_*CP*_ = 12.7, 8.7 Hz, py^6^), 139.6 (s, py^4^), 108.6 (vd, *J*_*CP*_ = 20.9 Hz, py^3^), 85.7 (vd, *J*_*CP*_ = 7.1 Hz, py^5^), 118.1 (py^3,5^), 39.4 (d, *J*_*CP*_ = 23.7 Hz, Cq), 38.2 (d, *J*_*CP*_ = 15.7 Hz, Cq), 28.5 (d, *J*_*CP*_ = 3.7 Hz, CH_3_), 27.9 (d, *J*_*CP*_ = 5.5 Hz, CH_3_) ppm; ^31^P{^1^H} NMR (101 MHz, C_6_D_6_, 20 °C): *δ* = 145.7 (A), 142.2 (B) (AB, *J*_*PP*_ = 84.5 Hz) ppm; IR (ATR): $\bar{\nu }$ = 1913 (*ν*_CO_), 1838 (*ν*_CO_) cm^−1^.

## X-ray structure determination

X-ray diffraction data of [Mn(PNP^NH^-*i*Pr)(CO)_3_]OTf (**3aOTf**), [Mn(PNP^CH2^-*i*Pr)(CO)_3_]Br·CH_2_Cl_2_ (**3dBr**·CH_2_Cl_2_), [Mn(PNP^NH^-*t*Bu)(CO)_2_]BF_4_ (**3eBF**_**4**_), [Re(PNP^NMe^-*i*Pr)(CO)_3_]Br·½acetone (**5bBr**·½acetone), and [Re(PNP^NH^-*t*Bu)(CO)_3_]Br (**5eBr**) (CCDC numbers 1865227-1865231) were collected at *T* = 100 K in a dry stream of nitrogen on a Bruker Kappa APEX II diffractometer system using graphite-monochromatized Mo-*Kα* radiation (*λ* = 0.71073 Å) and fine sliced *φ*- and *ω***-**scans. Data were reduced to intensity values with SAINT and an absorption correction was applied with the multi-scan approach implemented in SADABS or TWINABS [30]. The structures were solved by the dual-space approach implemented in SHELXT [31] and refined against *F*^2^ with SHELXL [32]. Non-hydrogen atoms were refined anisotropically. The H atoms connected to C atoms were placed in calculated positions and thereafter refined as riding on the parent atoms. The amine-Hs were located from difference Fourier maps and refined freely (**3eBF**_**4**_) or restrained to a N–H distance of 0.87 Å (**5eBr**). The Mn atoms and CO ligands in **3eBF**_**4**_ were refined as disordered about two positions. Contributions of disordered solvent molecules to the intensity data were removed for **5eBr** using the SQUEEZE routine of the PLATON [33] software suite. Molecular graphics were generated with the program MERCURY [34].

## Computational details

Calculations were performed using the Gaussian 09 software package [35] with the B3LYP functional without symmetry constraints, the Stuttgart/Dresden ECP (SDD) basis set to describe the electrons of the manganese and rhenium atoms and a standard 6-31G** basis for all other atoms as already described previously [36].

## Electronic supplementary material

Below is the link to the electronic supplementary material.

Supplementary material 1 (CIF 9996 kb)

## References

[1] Gorgas N, Kirchner K (2018). Acc Chem Res.

[2] Gorgas N, Kirchner K, Morales-Morales D (2018). Pincer Compounds: Chemistry and Applications.

[3] Garbe M, Junge K, Beller M (2017). Eur J Org Chem.

[4] Maji B, Barman M (2017). Synthesis.

[5] Kallmeier F, Kempe R (2018). Angew Chem Int Ed.

[6] Filonenko GA, van Putten R, Hensen EJM, Pidko EA (2018). Chem Soc Rev.

[7] Wei D, Roisnel T, Darcel C, Clot E, Sortais JP (2017). Chem Cat Chem.

[8] Vogt M, Nerush A, Iron M, Leitus G, Diskin-Posner Y, Shimon LJW, Ben-David Y, Milstein D (2013). J Am Chem Soc.

[9] Li H, Wei D, Bruneau-Voisine A, Ducamp M, Henrion M, Roisnel T, Dorcet V, Darcel C, Carpentier JF, Soule JF, Sortais JP (2018). Organometallics.

[10] Piehl P, Peña-López M, Frey A, Neumann H, Beller M (2017). Chem Commun.

[11] Mastalir M, Glatz M, Gorgas N, Stöger B, Pittenauer E, Allmaier G, Veiros LF, Kirchner K (2016). Chem Eur J.

[12] Bertini F, Glatz M, Gorgas N, Stöger B, Peruzzini M, Veiros LF, Kirchner K, Gonsalvi L (2017). Chem Sci.

[13] Tondreau AM, Boncella JM (2016). Polyhedron.

[14] Neumann J, Elangovan S, Spannenberg A, Junge K, Beller M (2017). Chem Eur J.

[15] Bruneau-Voisine A, Wang D, Roisnel T, Darcel C, Sortais JP (2017). Catal Commun.

[16] Glatz M, Stöger B, Kirchner K (2017). Acta Cryst.

[17] Radosevich AT, Melnick JG, Stoian SA, Bacciu D, Chen CH, Foman BM, Ozerov OV, Nocera DG (2009). Inorg Chem.

[18] Tondreau AM, Boncella JM (2016). Organometallics.

[19] Mukherjee A, Nerush A, Leitus G, Shimon LJW, Ben-David Y, Jalapa NAE, Milstein D (2016). J Am Chem Soc.

[20] Kulkarni NV, Brennessel WW, Jones WD (2018). ACS Catal.

[21] Fu S, Shao Z, Wang Y, Liu Q (2017). J Am Chem Soc.

[22] Rao KR, Korobkov I, Gabidullin B, Richeson D (2018). Polyhedron.

[23] Bruneau-Voisine A, Wang D, Dorcet V, Roisnel T, Darcel C, Sortais JB (2017). J Catal.

[24] Simoes JAM, Beauchamp JL (1990). Chem Rev.

[25] Perrin DD, Armarego WLF (1988). Purification of laboratory chemicals.

[26] Benito-Garagorri D, Becker E, Wiedermann J, Lackner W, Pollak M, Mereiter K, Kisala J, Kirchner K (2006). Organometallics.

[27] Öztopcu Ö, Holzhacker C, Puchberger M, Weil M, Mereiter K, Veiros LF, Kirchner K (2013). Organometallics.

[28] Salem H, Shimon LJW, Posner-Diskin Y, Leitus G, Ben-David Y, Milstein D (2009). Organometallics.

[29] Leung WP, Ip QWY, Wong SY, Mak TCW (2003). Organometallics.

[30] Bruker computer programs (2018). APEX2, SAINT, SADABS and TWINABS.

[31] Sheldrick GM (2015). Acta Crystallogr A.

[32] Sheldrick GM (2015). Acta Crystallogr C.

[33] Acta Spek AL (2009). Crystallogr.

[34] Macrae CF, Edgington PR, McCabe P, Pidcock E, Shields GP, Taylor R, Towler M, van de Streek J (2006). J Appl Cryst.

[35] Frisch MJ, Trucks GW, Schlegel HB, Scuseria GE, Robb MA, Cheeseman JR, Scalmani G, Barone V, Mennucci B, Petersson GA, Nakatsuji H, Caricato M, Li X, Hratchian HP, Izmaylov AF, Bloino J, Zheng G, Sonnenberg JL, Hada M, Ehara M, Toyota K, Fukuda R, Hasegawa J, Ishida M, Nakajima T, Honda Y, Kitao O, Nakai H, Vreven T, Montgomery JA, Peralta JE, Ogliaro F, Bearpark M, Heyd JJ, Brothers E, Kudin KN, Staroverov VN, Kobayashi R, Normand J, Raghavachari K, Rendell A, Burant JC, Iyengar SS, Tomasi J, Cossi M, Rega N, Millam JM, Klene M, Knox JE, Cross JB, Bakken V, Adamo C, Jaramillo J, Gomperts R, Stratmann RE, Yazyev O, Austin AJ, Cammi R, Pomelli C, Ochterski JW, Martin RL, Morokuma K, Zakrzewski VG, Voth GA, Salvador P, Dannenberg JJ, Dapprich S, Daniels AD, Farkas Ö, Foresman JB, Ortiz JV, Cioslowski J, Fox DJ (2009). Gaussian 09, revision A.02.

[36] Schröder-Holzhacker C, Gorgas N, Stöger B, Kirchner K (2016). Monatsh Chem.

